# Parent-offspring genotyped trios unravelling genomic regions with gametic and genotypic epistatic transmission bias on the cattle genome

**DOI:** 10.3389/fgene.2023.1132796

**Published:** 2023-04-06

**Authors:** Samir Id-Lahoucine, Joaquim Casellas, Filippo Miglior, Flavio S. Schenkel, Angela Cánovas

**Affiliations:** ^1^ Centre for Genetic Improvement of Livestock, Department of Animal Biosciences, University of Guelph, Guelph, ON, Canada; ^2^ Departament de Ciència Animal i dels Aliments, Universitat Autònoma de Barcelona, Barcelona, Spain

**Keywords:** transmission ratio distortion, epistasis, genotyped trios, Holstein, allelic and genotypic parameterizations

## Abstract

Several biological mechanisms affecting the sperm and ova fertility and viability at developmental stages of the reproductive cycle resulted in observable transmission ratio distortion (i.e., deviation from Mendelian expectations). Gene-by-gene interactions (or epistasis) could also potentially cause specific transmission ratio distortion patterns at different loci as unfavorable allelic combinations are under-represented, exhibiting deviation from Mendelian proportions. Here, we aimed to detect pairs of loci with epistatic transmission ratio distortion using 283,817 parent-offspring genotyped trios (sire-dam-offspring) of Holstein cattle. Allelic and genotypic parameterization for epistatic transmission ratio distortion were developed and implemented to scan the whole genome. Different epistatic transmission ratio distortion patterns were observed. Using genotypic models, 7, 19 and 6 pairs of genomic regions were found with decisive evidence with additive-by-additive, additive-by-dominance/dominance-by-additive and dominance-by-dominance effects, respectively. Using the allelic transmission ratio distortion model, more insight was gained in understanding the penetrance of single-locus distortions, revealing 17 pairs of SNPs. Scanning for the depletion of individuals carrying pairs of homozygous genotypes for unlinked loci, revealed 56 pairs of SNPs with recessive epistatic transmission ratio distortion patterns. The maximum number of expected homozygous offspring, with none of them observed, was 23. Finally, in this study, we identified candidate genomic regions harboring epistatic interactions with potential biological implications in economically important traits, such as reproduction.

## 1 Introduction

Transmission bias phenomenon (or transmission ratio distortion, TRD), defined as a departure from Mendelian expectations (Crow, 1999), is associated with a wide variety of mechanisms that affect the sperm and ova fertility and viability at developmental stages of the reproductive cycle (e.g., embryos, postnatal metabolism and growth, *etc.*). Although the genetic background of TRD is mainly related to single-locus factor (i.e., within a single gene or blocks of physically linked loci), interactions between different loci (i.e., epistasis) could also potentially cause specific TRD patterns (Corbett-Detig et al., 2013; Leppälä et al., 2013). Within a TRD context, compatible allele (or genotype) combinations at different loci increase fitness (Fickel and Weyrich, 2011), whereas unfavorable allelic (or genotype) combinations are under-represented, exhibiting deviation from Mendelian proportions (Montagutelli et al., 1996). In addition, as epistasis influences the expressivity of the involved loci, it has been hypothesized that the incomplete penetrance of lethal mutations may involve epistatic interaction (Ballinger and Noor, 2018).

There are documented cases in model organisms, plants and fish where epistatic interactions explain the variation of the observed TRD (Montagutelli et al., 1996; van Boven and Weissing, 2001; Borowsky and Cohen, 2013; Behrouzi and Wit, 2018). Epistatic TRD at different loci has been related to different sources, such as the lethality of embryos carrying particular combinations of alleles (Montagutelli et al., 1996) and lower fertility or diseases (Behrouzi and Wit, 2018), among others. Particularly, epistatic TRD has been related to genic incompatibilities (or hybrid incompatibility) in crosses where deleterious epistatic interactions between heterospecific alleles lead to hybrid sterility and/or inviability (Turelli and Orr, 2000; Fishman et al., 2008; Cutter, 2012; Corbett- Detig et al., 2013; Giesbers et al., 2019; Arends et al., 2022). These genic incompatibilities between diverged genomes, termed Bateson-Dobzhansky-Muller (BDM) incompatibilities, were first described by Bateson (1909), Dobzhansky (1937) and Muller (1942), representing a classic model for the evolution of reproductive isolation in diverging lineages.

Despite the additive paradigm being widely predominant for the technical statistical and computational limitations, the importance of interaction components of the genetic architecture of traits has recently been emphasized (Carlborg and Haley, 2004; Phillips, 2008). It must be highlighted that the relative importance of gene-by-gene interaction has been around for more than a century (Gilbert-Diamond and Moore, 2011) and was recognized as an explanation of the observed deviations from Mendelian ratios (e.g., Bateson, 1909; Neel and Schull, 1954; Hollander, 1955; Phillips, 1998). Identification of epistatic interaction in the genome will provide an opportunity to understand the epistatic effect on agronomically important complex traits allowing insights into the genetic background and complexity underlying reproduction. The TRD approach does not require phenotypic records, but only genomic information in trios (sire-dam-offspring), which is available nowadays for most of the livestock species what opens alternative genomic strategies with potential outcomes to improve reproductive success.

The conventional method of identifying epistatic TRD relies on performing a screen for pairs of loci showing deviations from the expected assuming random segregation and the majority of methods are restricted to an F2 design. The allelic and genotypic TRD models developed by Casellas et al. (2012, 2014, 2017) for single-locus, which are based on tracing allele inheritance from parents to offspring, are flexible and accommodate all types of matings and pedigree structure. Here we adapted these models to account for epistatic interactions and to be applicable for livestock populations. The main objectives of this research, using parent-offspring genotyped trios (sire-dam-offspring) of Holstein cattle, were to investigate the genetic basis for incomplete deviation of single-locus TRD and unraveling pairs of unlinked genomic regions across the whole genome that are not transmitted according to Mendelian inheritance rules, but display epistatic transmission ratio distortion patterns.

## 2 Materials and methods

### 2.1 Genotypes and trios

The dataset used to investigate epistatic TRD consisted of 436,651 Holstein genotypes provided by the Canadian Dairy Network database (Lactanet, Guelph, Ontario, Canada). The number of genotyped trios (sire-dam-offspring) was 283,817 and were sampled from all available Holstein genotypes (>1 million; October 2017) with offspring genotyped within 90 days of the birth, thus minimizing selection artifacts (Id-Lahoucine and Casellas, 2017; Id-Lahoucine et al., 2019a). The number of genotyped sire and dams was 5,224 and 117,316, respectively. Animals were genotyped with different SNP genotyping arrays ranging from 6,909 to 777,962 SNPs (Supplementary Material S1) and mapped to the UMD3.1 *Bos taurus* genome assembly. This data was previously imputed using FImpute (Sargolzaei et al., 2014) to 47,910 SNPs (Id-Lahoucine, 2020).

### 2.2 Analytical models statistical analyses

Epistatic transmission ratio distortion was evaluated on pairs of SNPs across the whole genome (47,910 SNPs) using three different methods. In order to avoid confounding due to linkage disequilibrium (LD) within chromosomes and to reduce the computational time, epistatic TRD scan was restricted to inter-chromosomal pairs.

***Genotypic parameterization of epistatic transmission ratio distortion.*** Following Casellas et al. (2012, 2020) genotypic TRD model of one single-locus and Kempthorne (1969) epistatic theory, epistatic TRD parameters were included in the probability of the offspring (P_off_) for each combination of genotypes of a given pair of two loci (A and B) as:

$$P_{off}\;\left(AABB\right)=1+1\;\alpha_{A}+1\;\alpha_{B}-1\;\delta_{A}-1\;\delta_{B}+1\;\alpha \alpha_{e}+1\;\alpha \delta_{e}+1\;\delta \alpha_{e}-1\;\delta \delta_{e}$$

$$P_{off}\;\left(AaBB\right)=2+0\;\alpha_{A}+1\;\alpha_{B}+1\;\delta_{A}-1\;\delta_{B}+0\;\alpha \alpha_{e}+0\;\alpha \delta_{e}-1\;\delta \alpha_{e}+1\;\delta \delta_{e}$$

$$P_{off}\;\left(aaBB\right)=1-1\;\alpha_{A}+1\;\alpha_{B}-1\;\delta_{A}-1\;\delta_{B}-1\;\alpha \alpha_{e}-1\;\alpha \delta_{e}+1\;\delta \alpha_{e}-1\;\delta \delta_{e}$$

$$P_{off}\;\left(AABb\right)=2+1\;\alpha_{A}+0\;\alpha_{B}-1\;\delta_{A}+1\;\delta_{B}+0\;\alpha \alpha_{e}-1\;\alpha \delta_{e}+0\;\delta \alpha_{e}+1\;\delta \delta_{e}$$

$$P_{off}\;\left(AaBb\right)=4+0\;\alpha_{A}+0\;\alpha_{B}+1\;\delta_{A}+1\;\delta_{B}+0\;\alpha \alpha_{e}+0\;\alpha \delta_{e}+0\;\delta \alpha_{e}-1\;\delta \delta_{e}$$

$$P_{off}\;\left(aaBb\right)=2-1\;\alpha_{A}+0\;\alpha_{B}-1\;\delta_{A}+1\;\delta_{B}+0\;\alpha \alpha_{e}+1\;\alpha \delta_{e}+0\;\delta \alpha_{e}+1\;\delta \delta_{e}$$

$$P_{off}\;\left(AAbb\right)=1+1\;\alpha_{A}-1\;\alpha_{B}-1\;\delta_{A}-1\;\delta_{B}-1\;\alpha \alpha_{e}+1\;\alpha \delta_{e}-1\;\delta \alpha_{e}-1\;\delta \delta_{e}$$

$$P_{off}\;\left(Aabb\right)=2+0\;\alpha_{A}-1\;\alpha_{B}+1\;\delta_{A}-1\;\delta_{B}+0\;\alpha \alpha_{e}+0\;\alpha \delta_{e}+1\;\delta \alpha_{e}+1\;\delta \delta_{e}$$

$$P_{off}\;\left(aabb\right)=1-1\;\alpha_{A}-1\;\alpha_{B}-1\;\delta_{A}-1\;\delta_{B}+1\;\alpha \alpha_{e}-1\;\alpha \delta_{e}-1\;\delta \alpha_{e}-1\;\delta \delta_{e}$$

where; α_A_ and δ_A_ are additive- and dominance-TRD parameters for locus A, respectively, α_B_ and δ_B_ are additive- and dominance-TRD parameters for locus B, respectively, αα_e_, αδ_e_, δα_e_ and δδ_e_ are additive-by-additive, additive-by-dominance, dominance-by-additive and dominance-by-dominance epistatic TRD parameters, respectively. The parametric space considered for all the parameters ranges from −1 to 1. Due to the complexity of this model and the number of parameters involved, negative probabilities must be rescaled to 0 for simplification. An additional restriction is needed to guarantee P_off_ (AABB) + P_off_ (AaBB) + P_off_ (aaBB) + P_off_ (AABb) + P_off_ (AaBb) + P_off_ (aaBb) + P_off_ (AAbb) + P_off_ (Aabb) + P_off_ (aabb) = 1. On the other hand, it must be emphasized that all the equations of (offspring) probabilities described here correspond to the unique case of heterozygous-by-heterozygous mating for both loci (i.e., AaBb × AaBb mating), and the probabilities needs to be adapted for each specific mating accordingly. For instance, the P_off_ (AABB) becomes 4 + 1 α_A_ + 1 α_B_ - 1 δ_A_ - 1 δ_B_ + 1 αα_e_ + 1 αδ_e_ + 1 δα_e_ - 1 δδ_e_ whereas P_off_ (AABb) is 0 for AaBB × AaBB mating. The total number of possible informative matings is 65 and which could be combined in 27 types of matings summarized in the Supplementary Material S2.

Under a Bayesian implementation, the conditional posterior probabilities of the TRD parameters are defined as:

$$p\left(\alpha_{A},\delta_{A},\alpha_{B},\delta_{B},\alpha \alpha_{e},\alpha \delta_{e},\delta \alpha_{e},\delta \delta_{e}\;\left|y)\propto p(y\right|\alpha_{A},\delta_{A},\alpha_{B},\delta_{B},\alpha \alpha_{e},\alpha \delta_{e},\delta \alpha_{e},\delta \delta_{e}\right)\;p\left(\alpha_{A}\right)\;p\left(\delta_{A}\right)\;p\left(\alpha_{B}\right)\;p\left(\delta_{B}\right)\;p\left(\alpha \alpha_{e}\right)\;p\left(\alpha \delta_{e}\right)\;p\left(\delta \alpha_{e}\right)\;p\left(\delta \delta_{e}\right)$$

where: **y** is the column vector of genotypes of the offspring generation.

In order to reduce the computational time, only pairs of SNPs with ≥1,000 informative offspring and ≥20 heterozygous sires or/and ≥50 heterozygous dams were used. Secondly, the observed and expected number of offspring for the 27 combined matings were determined for each pair of loci. The analyses included only pairs of SNPs with ≥1,000 under- or over-represented offspring for a specific two-locus genotype offspring and mating and this difference (between the observed and expected) being relatively 10% higher/lower than the expected number of offspring. Notice that these criteria and cut-off values were chosen based in the results observed in single-locus TRD analyses (Id-Lahoucine, 2020) and all these steps guaranteed a relevant deviation from Mendelian proportions in the corresponding regions allowing to target regions with potential epistatic TRD signals and reducing the computational time considerably. The model was implemented in a Bayesian approach with the metropolis-Hastings (Hastings, 1970) sampling technique. A preliminary scan was implemented with a unique Monte Carlo Markov chain of 11,000 iterations (the first 1,000 were discarded for TRD estimation). The statistical significance of TRD parameters was tested by a Bayes factor (BF, Kass and Raftery, 1995). More accurate estimates were obtained using 550,000 iterations for the selected relevant epistatic regions from the preliminary analyses. In addition, in order to focus mainly on epistasis phenomenon, the ratio of maximum (BF_αα_, BF_αδ_, BF_δα_, BF_δδ_)/maximum (BF_αA_, BF_δA_, BF_αB_, BF_δB_) > 1,000 was used to select the regions with more statistical evidence of epistatic effects rather direct effects. The BF was also used for the multiple test correction where the top 0.1% significant regions were selected as the most relevant epistatic TRD regions.

***Allelic parameterization of epistatic transmission ratio distortion.*** An allelic epistatic TRD could be targeted by tracing back the inheritance of a combination of two specific alleles of the two loci (i.e., an artificial haplotype) from parents to offspring. Thus, the probability of an offspring (P_off_) for each combination of genotypes of a given par of two loci (A and B) was parameterized to include both direct and epistatic allelic TRD effects as:

$$P_{off}\;\left(AABB\right)=\left[\left(0.5+\beta_{A}\right)\times \left(0.5+\beta_{B}\right)\times \left(0.5+\beta_{A}\right)\times \left(0.5+\beta_{B}\right)\right]\times \left(1+\beta_{AB/ab}\right)\times \left(1+\beta_{AB/ab}\right)$$

$$P_{off}\;\left(AAbb\right)=\left[\left(0.5+\beta_{A}\right)\times \left(0.5-\beta_{B}\right)\times \left(0.5+\beta_{A}\right)\times \left(0.5-\beta_{B}\right)\right]\times \left(1+\beta_{Ab/aB}\right)\times \left(1+\beta_{Ab/aB}\right)$$

$$P_{off}\;\left(aaBB\right)=\left[\left(0.5-\beta_{A}\right)\times \left(0.5+\beta_{B}\right)\times \left(0.5-\beta_{A}\right)\times \left(0.5+\beta_{B}\right)\right]\times \left(1-\beta_{Ab/aB}\right)\times \left(1-\beta_{Ab/aB}\right)$$

$$P_{off}\;\left(aabb\right)=\left[\left(0.5-\beta_{A}\right)\times \left(0.5-\beta_{B}\right)\times \left(0.5-\beta_{A}\right)\times \left(0.5-\beta_{B}\right)\right]\times \left(1-\beta_{AB/ab}\right)\times \left(1-\beta_{AB/ab}\right)$$

$$P_{off}\;\left(AABb,AAbB\right)=\left[\left(0.5+\beta_{A}\right)\times \left(0.5+\beta_{B}\right)\times \left(0.5+\beta_{A}\right)\times \left(0.5-\beta_{B}\right)\right]\times \left(1+\beta_{AB/ab}\right)\times \left(1+\beta_{Ab/aB}\right)$$

$$P_{off}\;\left(aaBb,aabB\right)=\left[\left(0.5-\beta_{A}\right)\times \left(0.5+\beta_{B}\right)\times \left(0.5-\beta_{A}\right)\times \left(0.5-\beta_{B}\right)\right]\times \left(1-\beta_{AB/ab}\right)\times \left(1-\beta_{Ab/aB}\right)$$

$$P_{off}\;\left(AaBB,aABB\right)=\left[\left(0.5+\beta_{A}\right)\times \left(0.5+\beta_{B}\right)\times \left(0.5-\beta_{A}\right)\times \left(0.5+\beta_{B}\right)\right]\times \left(1+\beta_{AB/ab}\right)\times \left(1-\beta_{Ab/aB}\right)$$

$$P_{off}\;\left(Aabb,aAbb\right)=\left[\left(0.5+\beta_{A}\right)\times \left(0.5-\beta_{B}\right)\times \left(0.5-\beta_{A}\right)\times \left(0.5-\beta_{B}\right)\right]\times \left(1-\beta_{AB/ab}\right)\times \left(1+\beta_{Ab/aB}\right)$$

$$P_{off}\;\left(AaBb,aAbB\right)=\left[\left(0.5+\beta_{A}\right)\times \left(0.5+\beta_{B}\right)\times \left(0.5-\beta_{A}\right)\times \left(0.5-\beta_{B}\right)\right]\times \left(1+\beta_{AB/ab}\right)\times \left(1-\beta_{AB/ab}\right)$$

$$P_{off}\;\left(AabB,aABb\right)=\left[\left(0.5+\beta_{A}\right)\times \left(0.5+\beta_{B}\right)\times \left(0.5-\beta_{A}\right)\times \left(0.5-\beta_{B}\right)\right]\times \left(1+\beta_{Ab/aB}\right)\times \left(1-\beta_{Ab/aB}\right)$$

where; β_A_ and β_B_ are direct TRD parameters for locus A and B, respectively, β_AB/Ab_, β_AB/aB_, β_AB/ab_, β_Ab/aB_, β_Ab/ab_ and β_aB/ab_ are the 6 heterozygous pairwise combinations for epistatic TRD parameters. Note that *AB*, *Ab*, *aB* and *ab* are the 4 possible artificial haplotype alleles of the two implicated SNPs. For all parameters, flat priors were assumed within a parametric space ranging from −0.5 to 0.5 for direct TRD effects and from −1.0 to 1.0 for epistatic TRD effects. The parametric space of direct effect is based on the principles of Mendelian inheritance (i.e., the probability of transmission of one specific allele ranges from 0 (e.g., β_A_ = −0.5) to 1 (e.g., β_A_ = 0.5), and where 0.5 (e.g., β_A_ = 0.0) corresponds to null TRD). In contrast, the range from −1 to 1 for epistatic parameters could be viewed as the positive/negative preferential transmission of one combination of two alleles (artificial haplotype) or its opposite heterozygous combinations, respectively, whereas 0 indicates null epistatic TRD. Also, these probabilities corresponded to the unique case of heterozygous-by-heterozygous mating (i.e., AaBb × AaBb), and the probabilities must be adapted for each specific mating accordingly.

Under a Bayesian implementation, the conditional posterior probabilities of the TRD parameters are defined as:

$$p\left(\beta_{A},\beta_{B},\beta_{AB/Ab},\beta_{AB/aB},\beta_{AB/ab},\beta_{Ab/aB},\beta_{Ab/ab},\beta_{aB/aB}\left|y)\propto p(y\right|\beta_{A},\beta_{B},\beta_{AB/Ab},\beta_{AB/aB},\beta_{AB/ab},\beta_{Ab/aB},\beta_{Ab/ab},\beta_{aB/aB}\right)\;p\left(\beta_{A}\right)\;p\left(\beta_{B}\right)\;p\left(\beta_{AB/Ab}\right)\;p\left(\beta_{AB/aB}\right)\;p\left(\beta_{AB/ab}\right)\;p\left(\beta_{Ab/aB}\right)\;p\left(\beta_{Ab/ab}\right)\;p\left(\beta_{aB/aB}\right)$$

where; **y** is the column vector of genotypes of the offspring generation.

This method is similar to the heterozygous pairwise combination procedure of haplotype analysis (single locus) described by Id-Lahoucine et al. (2019a), which is highly computationally demanding. For this reason, a simplified algorithm was implemented for a preliminary scan. The biallelic-haplotype procedure for haplotype analysis described by Id-Lahoucine et al. (2019a) was adapted to estimate TRD for the artificial haplotypes in order to target signals of epistasis. Thus, the transmission probability (P) of an artificial haplotype from heterozygote parents to offspring was parameterized including one overall epistatic TRD effect (α_j_) for each specific *j* artificial haplotype (AH) allele (P (AH_j_) = 1—P (AH_-j_) = 0.5 + α_j_).

As an exhaustive search for epistatic allelic TRD is computationally unfeasible, epistatic overall TRD were estimated only for the combination of pairs of SNPs which include one SNP with significant direct TRD effect already identified from analysis of single-locus TRD (2,962 SNPs; Id-Lahoucine, 2020). After the construction of artificial bi-allelic haplotypes, the analyses were performed within a Bayesian framework using a TRDscan v.1.0 software (Id-Lahoucine et al., 2019a) with a unique Monte Carlo Markov chain of 110,000 iterations where the first 10,000 iterations were discarded as burn-in. The statistical significance of TRD was evaluated using a Bayes factor (Kass and Raftery, 1995). Following this analysis, the candidate regions with signals of epistatic allelic pattern obtained by the simplified method were re-examined with the full epistatic allelic model (with 550,000 iterations), thus, allowing to overcome the limitation of this simplified method which was not parameterized to take into account both direct and epistatic effects simultaneously. Finally, both allelic and genotypic parameterizations were compared using the deviance information criterion (DIC, Spiegelhalter et al., 2002) to determine the goodness-of-fit and the epistatic pattern of each pair of regions.

***Double recessive epistatic transmission ratio distortion*.** The criteria applied for both genotypic and allelic model will discard the possibility of detecting double recessive epistatic TRD. In this sense, in order to target double recessive epistatic TRD, the biallelic-haplotype procedure with artificial haplotypes was implemented to screen for absent of double homozygous offspring for two-locus genotypes. For this, a minimal 15 none-observed offspring for one single combination of homozygous genotypes were chosen, while ensuring no deviation from Mendelian proportions for other genotypes.

*Multiple test correction*: For multiple test correction, the BF was used in order to choose the best candidate regions with epistatic effects. Thus, the multiple test correction was performed by selecting the top 0.1% of the regions within each category according to BF.

## 3 Results

### 3.1 Genotypic epistatic transmission ratio distortion

Pairs of regions with epistatic TRD estimates were found widely across the Holstein genome. Using a threshold of ≥1,000 for BF, a large number of pairs of SNPs were observed with decisive evidence for epistatic TRD. In order to discard false TRD and to identify the most relevant regions, the following steps were considered. Firstly, in order to focus on epistasis phenomenon, regions with more statistical relevance for direct effects than epistatic effects were discarded. Thus, only region with a minimal ratio of 1,000 between the maximum BF of epistatic and direct effects were considered (i.e., maximum (BF_αα_, BF_αδ_, BF_δα_, BF_δδ_)/maximum (BF_αA_, BF_δA_, BF_αB_, BF_δB_) > 1,000). Secondly, estimates with large credible intervals were discarded as artifacts of the convergence of the model following Id-Lahoucine (2020). For this purpose, pairs of SNPs with a coefficient of variation >20% for significant epistatic effects were excluded. Notice that from single-locus TRD, when clear TRD exist, the standard variation of TRD estimates is mostly null (<0.01), thus, the use of coefficient of variation of TRD estimates is a straightforward rule to discard regions with instable convergence. Thirdly, given that most regions have several direct and epistatic effects with different statistical significance, the results were separated into their corresponding effects (i.e., additive-by-additive, additive-by-dominance/dominance-by-additive or dominance-by-dominance) according to the most relevant effect in terms of BF. After filtering the results following the previous steps, the number of the obtained pairs were 59,831, 87,699 and 20,549 for additive-by-additive, additive-by-dominance/dominance-by-additive and dominance-by-dominance effects, respectively.

After implementing a multiple test correction, which was based on selecting the top 0.1% of the regions within each category according to BF, the number of regions reduced to 169, being 60, 88 and 21 with additive-by-additive, additive-by-dominance/dominance-by-additive and dominance-by-dominance effects, respectively. This strategy was implemented with the objective to select the most significant “top hits” of epistatic TRD signals following François et al. (2016) and Boschiero et al. (2018). These pairs of regions were chosen for a second analysis with a large Monte Carlo Markov chain of 500,000 iterations. Applying the previous criteria described above to the new estimated, 55 out of 88 regions with additive-by-dominance/dominance-by-additive were discarded. Moreover, among the identified pairs, we observed that some SNPs interacted with more than one individual SNP. For instance, the SNP BTA17:39,696,262 interacted with 26 SNPs covering from 4,197,354 bp to 12,447,484 bp on BTA23. Within this context, only the pair of SNPs with the highest BF were maintained as the best candidate regions when one single locus interacts with several physically linked SNPs. Thus, the number of regions reduced to 7, 19 and 6 with additive-by-additive, additive-by-dominance/dominance-by-additive and dominance-by-dominance effects, respectively. These results are summarized in Table 1 for additive-by-additive and dominance-by-dominance effects and in Table 2 for additive-by-dominance/dominance-by-additive. Additional details (e.g., number of informative parents, frequencies, *etc.*) are presented in the Supplementary Material S3.

**TABLE 1:** Pairs of genomic regions identified with epistatic transmission ratio distortion (TRD) in Holstein cattle with mainly additive-by-additive or dominance-by-dominance effects.

Coordinates locus A and B	α_A_ ^a^ (Log_10_(BF^b^))	α_B_ (Log_10_(BF))	δ_A_ (Log_10_(BF))	δ_B_ (Log_10_(BF))	αα_e_ (Log_10_(BF))	αδ_e_ (Log_10_(BF))	δα_e_ (Log_10_(BF))	δδ_e_ (Log_10_(BF))	Log_10_ (LR^c^)	DIC^d^ genotypic model	DIC allelic model
1:41490266 × 21:26405185	0.15 (693.7)	−0.04 (22.8)	0.43 (1524.4)	0.44 (1756.7)	1 (6794.4)	−0.22 (850)	−0.03 (9.9)	−0.94 (6459.6)	12033	269451.4	283614.8
1:95213011 × 23:30121507	−0.06 (41.4)	−0.29 (1358.8)	0.44 (2169.2)	0.33 (853.2)	−1 (5112.1)	−0.07 (46.3)	0.42 (1663.7)	−0.88 (4899.6)	10342.8	243497.0	265045.6
4:85759993 × 21:37978844	0.38 (1191.4)	−0.24 (776.9)	0.22 (366.2)	0.29 (639.9)	1 (4681.5)	−0.43 (1498.1)	0.25 (736.4)	−0.64 (2876.6)	7971.1	272790.3	278502.2
10:20146107 × 14:29246158	0.3 (1014.1)	−0.06 (397.4)	0.33 (915.8)	0.42 (1846.2)	1 (5903.5)	−0.4 (1748.2)	−0.01 (−0.1)	−0.82 (4587)	10775.9	266355.8	278134.6
17:39696262 × 23:5524345	0.05 (24.5)	−0.27 (1239.7)	0.46 (3596)	0.4 (1365.5)	1 (6648.6)	0.06 (73.2)	0.37 (1619.3)	−0.94 (6250.2)	12782.1	268513.8	283693.9
21:60175026 × 27:1343227	0 (−1.5)	0.18 (896.7)	0.34 (862.5)	0.3 (670.5)	1 (4880.7)	−0.05 (23.6)	−0.23 (889.3)	−0.74 (3597.7)	8058.9	264658.6	276906.1
23:1674622 × 28:2233574	0.57 (605.3)	−0.67 (759.9)	−0.36 (450)	−0.6 (1355.6)	−0.99 (5388.6)	0.51 (598.9)	−0.44 (410.9)	−0.18 (114.5)	5117	215691.1	262136.3
1:36209316 × 21:26405185	0.26 (1526.4)	0.14 (756.9)	0.48 (1906.4)	0.47 (9303.7)	1 (10097.7)	−0.34 (2110.8)	−0.24 (1429.7)	−1 (16219.5)	25721.6	235138.9	272410.9
1:95101691 × 23:29897088	0.09 (66.9)	−0.06 (23.7)	0.39 (1362.2)	0.33 (979.3)	1 (5376.7)	0.11 (111)	0.21 (829.7)	−1 (13060.3)	15124.9	239767.8	268110.2
4:85384769 × 21:37978844	0.45 (3133.8)	0.33 (2055.7)	0.4 (4817.4)	0.47 (4527.9)	1 (9437.1)	−0.54 (3956.6)	−0.49 (3653.7)	−1 (20223.3)	32514.9	202493.3	252222.1
10:25732248 × 14:29246158	−0.18 (863.3)	0.44 (2600)	0.58 (6551.3)	0.39 (9252)	−1 (9299.1)	0.33 (1901.5)	−0.62 (4533.1)	−1 (15595.4)	27517.3	214749.7	257410.0
13:1655502 × 28:21945694	−0.1 (49.7)	0.17 (159.4)	0.14 (148.6)	0.19 (240.6)	0.8 (1708.3)	0.13 (96.1)	−0.11 (76.8)	−0.59 (8447)	3613.9	267493.0	279825.4
17:39696262 × 23:6727000	0.2 (950)	−0.37 (1855.9)	0.51 (8181.6)	0.38 (1435.3)	1 (7685.7)	−0.1 (75.5)	0.52 (2916.7)	−1 (10688.5)	18451.2	247479.4	276381.9

^a^ α_A_ and δ_A_ are additive- and dominance-TRD, parameters for locus A, respectively, α_B_ and δ_B_ are additive- and dominance-TRD, parameters for locus B, respectively, αα_e_, αδ_e_, δα_e_ and δδ_e_ are additive-by-additive, additive-by-dominance, dominance-by-additive and dominance-by-dominance epistatic TRD, parameters, respectively.

^b^ Bayes Factor.

^c^ Likelihood ratio [= L (**y** | α_A_, δ_A_, α_B_, δ_B_, αα_e_, αδ_e_, δα_e_, δδ_e_)/L (**y** | α_A_ = 0, δ_A_ = 0, α_B_ = 0, δ_B_ = 0, αα_e_ = 0, αδ_e_ = 0, δα_e_ = 0, δδ_e_ = 0)].

^d^ Deviance information criterion.

**TABLE 2:** Pairs of genomic regions identified with epistatic transmission ratio distortion (TRD) in Holstein cattle with mainly additive-by-dominance effects.

Coordinates locus A and B	α_A_ ^a^ (Log_10_(BF^b^))	α_B_ (Log_10_(BF))	δ_A_ (Log_10_(BF))	δ_B_ (Log_10_(BF))	αα_e_ (Log_10_(BF))	αδ_e_ (Log_10_(BF))	δα_e_ (Log_10_(BF))	δδ_e_ (Log_10_(BF))	Log_10_ (LR^c^)	DIC^d^ genotypic model	DIC allelic model
1:33593708 × 21:26405185	0.43 (357)	−0.71 (1273.1)	0.11 (751.5)	−0.12 (670.2)	1 (1443.4)	−0.73 (1052.1)	0.92 (1917.7)	−0.52 (1220.9)	5319.8	221678.0	219188.4
1:44623201 × 7:10835967	0.72 (3173.9)	0.02 (0)	−0.15 (184.7)	0.64 (2276.9)	0.03 (3.5)	−0.47 (4339.9)	0.02 (0.8)	−0.07 (36.9)	3628.4	246495.6	264314.5
2:27724930 × 7:10835967	0.4 (1073.9)	0.03 (2.1)	−0.04 (8.1)	0.67 (1566.7)	0.04 (3.6)	−0.52 (1646.3)	−0.01 (−1.4)	0 (−1.3)	2753.3	209389.9	191723.9
4:18207858 × 7:10835967	−0.52 (1757.5)	0.08 (44.2)	−0.07 (38.7)	0.7 (2655.6)	−0.02 (0.5)	0.36 (2845.3)	−0.03 (4.4)	−0.1 (82.4)	3372	247299.9	261595.1
4:85188654 × 21:37978844	0.23 (42.4)	0.67 (1067.4)	0.57 (1730.8)	0.12 (89.5)	−1 (1473)	−0.27 (122.7)	−0.93 (2212.7)	−0.77 (2208.7)	5816.1	176258.6	178503.0
5:2793760 × 17:72942592	−0.9 (2033.2)	0.15 (88.8)	−0.2 (276.7)	0.29 (359.8)	−0.21 (128.9)	0.3 (2082.9)	0.03 (1.4)	−0.01 (−1.1)	2352.4	237918.0	250837.9
5:56169416 × 7:10835967	−0.5 (1508.3)	0.06 (12.1)	0 (−1.4)	0.63 (1429)	−0.06 (7.6)	0.56 (1931.3)	−0.02 (−0.4)	−0.06 (19.8)	2519.5	220619.4	212445.1
6:4076731 × 7:10835967	0.52 (1493.3)	0.06 (23.6)	−0.09 (66.8)	0.65 (2364.1)	0.03 (3.4)	−0.35 (3647.7)	−0.02 (0.4)	−0.08 (51.6)	3048.1	238330.7	258680.7
7:10835967 × 8:101251865	0.02 (0.8)	−0.46 (787.9)	0.69 (1468.1)	0 (−1.1)	−0.02 (−0.2)	0 (−1.4)	0.62 (1545.9)	−0.04 (6)	2833.8	199930.3	179384.2
7:10835967 × 10:29761646	0.07 (29.3)	0.69 (3299.7)	0.67 (2574.4)	−0.19 (361.4)	0.04 (7.2)	−0.01 (−1.5)	−0.44 (3652.7)	−0.04 (14.5)	3957.7	256869.6	272963.3
7:10835967 × 11:77484457	0.06 (22.9)	0.58 (2209)	0.64 (2631.8)	−0.06 (27.9)	0.03 (3.1)	−0.02 (0.1)	−0.38 (4236.9)	−0.11 (117)	3438.7	262239.4	281357.1
7:10835967 × 16:34911228	0 (−1.4)	0.38 (889.3)	0.68 (1568.8)	−0.02 (−0.1)	0.04 (5.9)	0 (−1.4)	−0.57 (1609.4)	−0.05 (16.3)	2815.2	207906.1	187295.0
7:10835967 × 17:48671784	0.05 (10.8)	0.42 (1673.5)	0.63 (1491.3)	−0.07 (24.1)	0.03 (0.8)	−0.01 (−1)	−0.53 (1737.3)	0.01 (−1.3)	2669.2	218822.9	207813.4
7:10835967 × 21:44499384	0.06 (15.4)	−0.37 (1084.4)	0.66 (1537.6)	0 (−1.3)	−0.01 (−0.6)	−0.02 (1.1)	0.51 (1486.6)	−0.03 (5.1)	2644.1	211959.4	192902.4
7:10835967 × 24:33863680	0.02 (0.3)	−0.37 (1189.2)	0.68 (1673)	−0.01 (−0.3)	−0.03 (2.6)	−0.01 (−1)	0.51 (1559.1)	−0.05 (11.8)	2755.5	210855.8	194410.1
8:76620508 × 25:28413439	−0.47 (44.8)	−0.97 (477.8)	−0.15 (16.7)	0.43 (180.9)	0.06 (4.1)	0.74 (1663.6)	−0.18 (20.3)	0.3 (99)	551.9	112941.5	105642.5
9:59595970 × 18:28139889	−0.57 (36.8)	−0.78 (313.5)	−0.31 (35.2)	0.48 (212.2)	0.39 (114.6)	0.84 (1223.8)	−0.35 (75.2)	0.5 (246.6)	247.6	122457.6	111722.0
10:26700563 × 14:29246158	−0.49 (455.7)	−0.62 (898.8)	−0.03 (13.5)	−0.1 (550.8)	−1 (1667.1)	0.85 (1627.5)	0.88 (1964.1)	−0.5 (1132.9)	5973.3	235802.2	234589.0
15:82260685 × 18:10642175	−0.55 (1299.8)	0.18 (105)	0.04 (8.6)	0.04 (6.1)	0.72 (1109.4)	0.23 (305.8)	−0.33 (2251.6)	−0.25 (608.7)	2261.2	258076.8	270979.5

^a^ α_A_ and δ_A_ are additive- and dominance-TRD, parameters for locus A, respectively, α_B_ and δ_B_ are additive- and dominance-TRD, parameters for locus B, respectively, αα_e_, αδ_e_, δα_e_ and δδ_e_ are additive-by-additive, additive-by-dominance, dominance-by-additive and dominance-by-dominance epistatic TRD, parameters, respectively.

^b^ Bayes Factor.

^c^ Likelihood ratio (= L (**y** | α_A_, δ_A_, α_B_, δ_B_, αα_e_, αδ_e_, δα_e_, δδ_e_)/L (**y** | α_A_ = 0, δ_A_ = 0, α_B_ = 0, δ_B_ = 0, αα_e_ = 0, αδ_e_ = 0, δα_e_ = 0, δδ_e_ = 0)).

^d^ Deviance information criterion.

When comparing between the genotypic and allelic models, different goodness-of-fit values were observed among the regions with additive-by-additive effects displaying reductions from 1,186.35 to 46,445.15 DIC units favoring the genotypic model over the allelic model. Notice that models with smaller DIC values indicate a better fit, and differences between models greater than 3 DIC units are considered statistically relevant (Spiegelhalter et al., 2002). In the case of regions with dominance-by-dominance effects, reductions up to 49,728.74 DIC units were observed. In addition, out of the regions found with additive-by-dominance/dominance-by-additive, only 8 pairs of regions favored the genotypic model with differences of up 20,349.96 DIC units. Therefore, the remaining pairs of regions (11) detected with the genotypic model with additive-by-dominance/dominance-by-additive showed better goodness-of-fit with the allelic model in terms of DIC. Nevertheless, more statistical relevance based on BF was observed for TRD parameters of genotypic model (up to log_10_(BF) = 1,964.13) in comparison to TRD estimated of allelic model (up to log_10_(BF) = 269.55). It is important to remember that DIC was computed based on all TRD parameters combined and the regions here were separated and selected based on their type of effects (e.g., additive-by-additive, additive-by-dominance) and BF.

### 3.2 Allelic epistatic transmission ratio distortion

Using the simplified method of the allelic model, a minimal increase of 0.05 in the TRD magnitude explained by the artificial haplotype (i.e., gamete) compared to the single SNP and an equal or greater number of under-/over-represented offspring (i.e., ≈ α_j_ × 2 × number of informative offspring) were considered to identify candidate regions with allelic epistatic TRD pattern. The total number of obtained regions detected with BF ≥ 1,000 was 4,852. For the identified artificial haplotypes, the absolute TRD magnitude ranged from |0.06| to |0.42|, and the maximum number of under- and/or over-represented offspring was 12,756 genotypes. The additional magnitude of TRD explained by artificial haplotypes reached a maximum of 0.25, with 8, 166 and 569 pairs with 0.20, 0.15 and 0.10, respectively. For example, pairs from the top regions (with highest BF) were found with individual SNP (BTA23:6,948,746) displaying an overall TRD of −0.22 and 3,078 under-represented offspring (log_10_(BF) = 302.62) and exhibited a deviation of −0.37 and 3,908 under-represented offspring (log_10_(BF) = 692.38) when paired with a specific allele in another SNP (BTA1:45,157,959). Thus, when the same SNP was found interacting in several pairs, we considered the pair of loci with the highest BF as the best candidate region with epistatic TRD. This reduced the number of pairs of regions to 78 (Supplementary Material S4). In terms of goodness-of-fit, among the 78 pairs of regions detected with the allelic model, minimal DIC values were observed on 76 pairs when compared to the genotypic model. Specifically, reduction from 19.38 up to 23,647.43 DIC units were observed for the allelic model in comparison to the genotypic model. The re-analyses of these regions with the full allelic model, with epistatic and direct TRD effects, displayed significant epistatic TRD effect with log_10_(BF) > 9.61, being log_10_(BF) = 969.06 the maximum value observed for an epistatic heterozygous pairwise combination. The number of regions that showed clear epistatic effects with the full model in all heterozygous pairwise combinations including the artificial haplotype were 29 and reduced to 17 when only one single artificial haplotype displayed epistatic TRD (Table 3).

**TABLE 3:** Pairs of genomic regions identified with epistatic transmission ratio distortion (TRD) in Holstein cattle with allelic patterns.

Coordinates locus A	Coordinates locus B	Artificial haplotype^a^	overall-TRD for AH (Log_10_(BF^b^))	β_A_ ^c^ (Log_10_(BF))	β_B_ (Log_10_(BF))	β_AB/Ab_ (Log_10_(BF))	β_AB/aB_ (Log_10_(BF))	β_AB/ab_ (Log_10_(BF))	β_Ab/aB_ (Log_10_(BF))	β_Ab/ab_ (Log_10_(BF))	β_aB/ab_ (Log_10_(BF))	DIC^d^ allelic Model	DIC genotypic Model
1:26453228	10:29751813	aB	−0.13 (54.4)	0.03 (3.7)	0 (−2)	0.01 (−1.7)	0.08 (1.6)	−0.31 (42.7)	0.33 (41.6)	0 (−0.9)	−0.4 (0.3)	119791.6	135155.3
1:39339779	7:94639822	Ab	−0.18 (105.4)	0 (−2)	0.05 (14.7)	0.47 (58.3)	0 (−1.7)	−0.27 (35.4)	−0.17 (11.6)	0 (0)^4^	−0.07 (1.8)	135191.8	156489.9
1:45157959	23:6948746	Ab	−0.37 (692.4)	0.14 (98.3)	0.22 (302.6)	0.73 (693.5)	0.28 (90.2)	0 (0)	0 (0)	0 (0)	0 (0)	5093.482	5112.857
2:16177570	12:38409372	aB	−0.27 (104.7)	0 (−2)	−0.12 (38.6)	−0.09 (0.3)	−0.5 (0.4)	0.3 (13.2)	0.34 (15.8)	0 (−1.7)	−0.58 (57.6)	118348.2	134441.9
2:116064542	19:15841374	ab	−0.17 (107.7)	0.01 (4.3)	0.01 (3.7)	0 (−1.7)	−0.01 (−1.2)	0.1 (5.5)	−0.14 (11.3)	0.92 (236.3)	0 (-0.5)	71487.01	73503.76
3:115888900	15:41707747	aB	−0.09 (109.5)	0.02 (19.5)	−0.01 (1.9)	0 (−1.7)	0.14 (16.5)	−0.14 (40.5)	0.09 (15.2)	−0.01 (-1)	−0.05 (-0.7)	151586.2	161556.2
4:32529693	13:37865729	AB	−0.26 (255.6)	−0.1 (62.8)	0 (−2)	0.57 (0.7)	−0.57 (117.2)	−0.36 (50.2)	0.38 (63.9)	−0.02 (−1)	0 (−1.7)	134058.1	155799.7
5:52771740	7:23568940	ab	−0.11 (81)	0 (−2)	0.04 (17.8)	−0.05 (−0.3)	0 (−1.7)	0.17 (15.6)	0.25 (42.9)	0.01 (0)	0.08 (5)	134919.9	154360.6
5:73713772	12:5817779	ab	−0.4 (313.7)	0.23 (149.1)	0 (−2)	−0.01 (−1.7)	0.25 (4.1)	0.77 (216)	−0.22 (8.5)	0.05 (-0.3)	0.51 (0.4)	129106.5	149538.6
5:106734118	8:9600065	AB	−0.17 (78.9)	−0.07 (20.7)	0 (−2)	0.08 (-0.4)	−0.31 (27.6)	−0.29 (24.4)	0.27 (21.7)	0.04 (−0.7)	0.01 (−1.7)	110051.4	123003.1
7:76349643	11:77560928	aB	−0.12 (135.7)	0.06 (47.6)	0 (−1.9)	0 (−1.7)	0.1 (10.9)	−0.21 (52.2)	0.23 (59.6)	0.18 (6.4)	0 (-0.2)	137400.3	154216.9
8:33964937	21:19601568	Ab	−0.12 (54)	0 (−2)	0.02 (2.7)	0.12 (3.7)	0 (−1.7)	−0.21 (28.2)	−0.2 (22)	−0.2 (0.1)	−0.09 (6.8)	136738.1	156144.7
8:49283003	10:95891436	Ab	−0.1 (39.5)	0 (−2)	0.05 (12.1)	0.12 (8.4)	0 (−1.7)	−0.22 (14.8)	−0.31 (28.7)	−0.6 (0.6)	−0.01 (−0.9)	104062.9	114919.1
9:44932317	27:30050354	ab	0.27 (99)	0.04 (7.6)	−0.01 (−0.4)	0 (−1.7)	0.06 (1.3)	−0.57 (107.4)	0.62 (137.9)	0.09 (-0.4)	−0.6 (0.6)	73645.82	77756.45
9:86330663	24:9732103	Ab	−0.1 (122.8)	−0.01 (4.3)	0.04 (37.8)	0.3 (88.9)	−0.01 (−1.7)	−0.13 (25.8)	−0.04 (1.5)	0.03 (-0.6)	−0.04 (1.8)	148273.6	166832.4
13:18832047	29:48033872	aB	−0.24 (54.3)	0 (−2)	−0.14 (30.9)	0.02 (−0.9)	0 (0)	−0.04 (−0.8)	0.08 (-0.4)	0 (−1.7)	−0.53 (39.2)	129725.9	149664.9
16:24948459	21:42668952	ab	−0.18 (46.6)	0.07 (10.7)	0 (−2)	−0.01 (−1.7)	0.07 (−0.6)	0.43 (40.9)	−0.26 (12.3)	0.13 (1.5)	−0.01 (0.1)	111212.7	125056.7

^a^ Biallelic-haplotype method.

^b^ Bayes Factor.

^c^ βA and β_B_ are direct TRD, parameters for locus A and B, respectively, β_AB/Ab_, β_AB/aB_, β_AB/ab_, β_Ab/aB_, β_Ab/ab_ and β_aB/ab_ are the 6 heterozygous pairwise combinations for epistatic TRD, model.

^d^ Deviance information criterion.

### 3.3 Recessive epistatic transmission ratio distortion

The total number of pairs of regions with at least 15 expected homozygous offspring but none of them observed assuming random assortment for both implicated SNPs, was 67. This was reduced to 56 after discarding pairs of regions pointing to physically related regions (Table 4, Supplementary Material S5). The maximum number of expected homozygous offspring for both SNPs, but none of them observed, was 23. The number of informative sires, dams and offspring for the identified regions reached to 105, 2,809 and 11,995, respectively.

**TABLE 4:** Pairs of genomic regions identified with epistatic transmission ratio distortion (TRD) in Holstein cattle with recessive patterns.

Coordinates locus A	Coordinates locus B	Artificial haplotype allele (genotype)	number of heterozygous sires	number of heterozygous dams	Frequency	Number of unobserved homozygous offspring	hH x hh^a^		hH x HH		hH x hH		
							Hh^b^	hH	hH	HH	hh	hH	HH
5:10278129	27:25728096	aB (aaBB)	105	2642	0.01	23	0	0	4382	4878	0	41	23
9:93731471	21:19601568	Ab (AAbb)	70	2296	0.009	22	0	0	4462	4626	0	34	22
11:32153221	21:19601568	ab (aabb)	68	1961	0.009	19	0	0	4155	4557	0	29	19
24:39650461	29:28523337	aB (aaBB)	80	2688	0.008	18	1	0	3337	3648	0	36	18
15:5766975	23:24441569	Ab (AAbb)	65	1650	0.007	18	0	0	3509	3619	0	28	18
15:54803685	16:79553324	Ab (AAbb)	59	1841	0.007	18	0	0	3583	3671	0	28	18
7:4436640	13:72570537	aB (aaBB)	72	2524	0.008	18	0	0	3598	3770	0	27	18
11:30575025	25:4667894	Ab (AAbb)	71	2506	0.009	18	0	0	3720	3792	0	29	18
8:82622674	20:65064787	Ab (AAbb)	102	1415	0.011	18	0	0	5954	5991	0	32	18
4:84118706	10:29375348	AB (AABB)	71	2744	0.009	17	0	0	3579	3909	0	31	17
1:86931672	16:12741497	ab (aabb)	69	1183	0.006	17	0	0	3835	3965	0	27	17
1:7782816	9:81277154	ab (aabb)	94	2809	0.009	17	0	0	4033	4274	0	30	17
7:26984291	12:18804912	Ab (AAbb)	70	1535	0.009	16	0	1	2638	2839	0	24	16
14:82345678	20:33773531	ab (aabb)	65	2504	0.01	16	0	0	2630	2874	0	24	16
6:47791866	25:34622211	Ab (AAbb)	66	2548	0.007	16	0	0	2925	3044	0	26	16
14:20606100	17:19346381	ab (aabb)	62	1998	0.007	16	0	0	3168	3284	0	25	16
9:7602607	10:57131487	aB (aaBB)	67	1900	0.007	16	0	0	3207	3399	0	24	16
10:44445350	17:19346381	aB (aaBB)	65	2085	0.007	16	0	0	3297	3399	0	25	16
15:62512689	16:79449472	AB (AABB)	58	1710	0.007	16	0	0	3511	3597	0	24	16
3:30194236	25:20619710	Ab (AAbb)	39	1595	0.006	16	0	0	3497	3630	0	27	16

^a^ The carrying status of the artificial haplotype in the parents’ generation.

^b^ The carrying status of the artificial haplotype in the offspring generation; h: artificial haplotype; H: alternative haplotypes.

## 4 Discussion

The current accessibility of high-throughput genotyping technologies in big data era have allowed the investigation of epistasis more deeply. Previous studies used genotype data and phenotypes or gene expression to investigate epistatic interactions between loci (e.g., Strange et al., 2013; Hemani et al., 2014; Huang et al., 2014; Mackay, 2014). Nevertheless, though many efforts have been done for identifying epistatic interactions, the methods/algorithmics are still being developed (e.g., Beissinger et al., 2015; Sun et al., 2016; 2017; Behrouzi and Wit, 2018; Id-Lahoucine et al., 2019b). In this paper we extended TRD models from single-locus to capture epistatic interactions, presenting a practical methodology when trios of parent-offspring genotypes (sire-dam-offspring) are available, not being restricted to only F2 designs, which is the most commonly used design in recent studies of TRD and epistasis in model organisms (e.g., Brennan et al., 2014; Niedzicka et al., 2017; Haddad et al., 2018).

### 4.1 Genotypic epistatic transmission ratio distortion

The Bayes factor is the standard Bayesian tool to compare two competing models (Kass and Raftery, 1995). For determining statistical significance, the possible choices of a BF threshold could be variable, BF ≥ 10 indicating strong evidence and BF ≥ 100 more decisive evidence according to the Jeffreys’ (1984) scale. Within the context of TRD analysis, the BF can be viewed as a measure of the strength of statistical evidence of the detected regions, given both TRD magnitude and sample size of informative offspring simultaneously. The value of BF increases with the increase of TRD magnitude and the number of informative offspring (Id-Lahoucine et al., 2019a). Here, a higher threshold of BF (≥1,000) was considered to ensure targeting epistatic regions with relevant TRD magnitudes. Even though the prevalence of signals of epistatic TRD widely extended across the Holstein genome (i.e., a large number of pairs of SNPs (>150,000) were initially identified), it is expected that part of them are simply artifacts of the sampling fluctuations that generate random/false TRD. It should be noted that the complexity and dimensionality of the model itself with 8 parameters and their possible interactions in the same data could generate false epistatic TRD. It was previously reported that the structure of the data with an unbalanced number of trios across matings could easily generate TRD artifacts (Casellas et al., 2020; Id-Lahoucine, 2020). For example, an inappropriate convergence of TRD estimation is produced when combinations of different TRD parameters maximize the likelihood of the data and result in large credible intervals for TRD estimates (including often zero values). For this reason, a minimal dispersion of TRD effects was assumed to discard potential TRD artifacts.

Given the scope of the study, which focus mainly on epistasis phenomenon, the ratio of maximum (BF_αα_, BF_αδ_, BF_δα_, BF_δδ_)/maximum (BF_αA_, BF_δA_, BF_αB_, BF_δB_) was used to select the regions with more statistical evidence of epistatic effects rather direct effects. Assuming, for example, that BF_αα_ [= p (αα_e_ = 0)/p (αα_e_ = 0|y)] and BF_αA_ [= p (α_A_ = 0)/p (α_A_ = 0|y)] are the maximum values, this ratio becomes = p (α_A_ = 0|y)/p (αα_e_ = 0|y). Given p (α_A_ = 0|y) = (p (y|α_A_ = 0)p (α_A_ = 0))/p(y), the ratio simplifies to p (y|α_A_ = 0)/p (y|αα_e_ = 0), a simple and direct measure of comparison between direct and epistatic effects. Thus, by using a ratio of 1,000, we ensured that the epistatic effect is much more relevant to explain the observed data of the implicated regions than the direct effects.

After the multiple test correction (top 0.1% of the regions within each category according to BF), the minimal and maximal log_10_(BF) obtained after the multiple test correction was 1,223.8 and 20,223, respectively. Notice that BF is a measure of the strength of statistical evidence (for both TRD magnitude and sample size of informative offspring). The likelihood of the data assuming epistatic effects for these regions is ≥ 10^1223.8^ times more probable that assuming null TRD effects, very strongly supporting the presence of epistatic effects.

**FIGURE 1:**
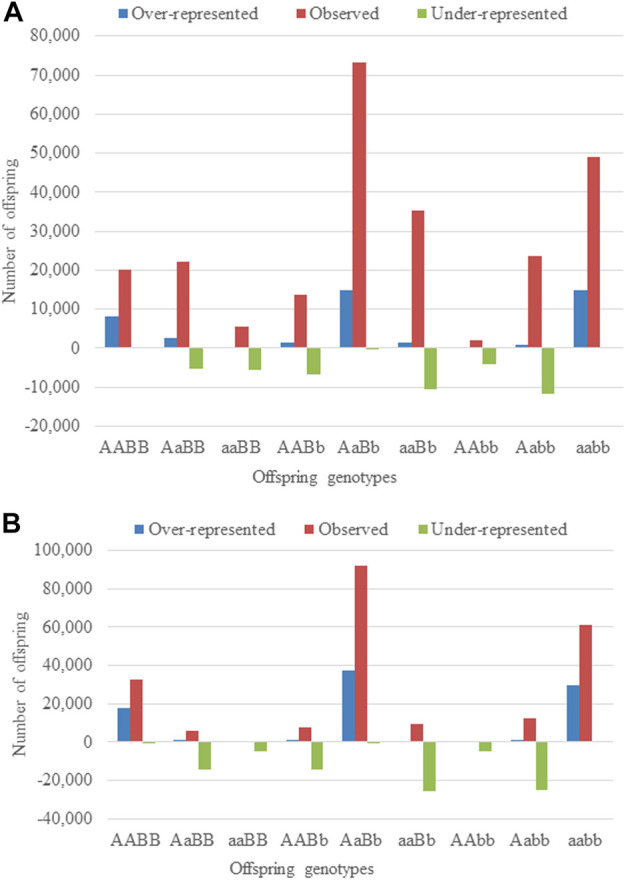
The observed, under- and over-represented offspring across matings in two-locus genotype with **(A)** additive-by-additive and **(B)** dominance-by-dominance epistatic effects.

It is important to mention that while the number of regions with additive-by-additive and dominance-by-dominance did not reduce after using large sample chains, they were considerably reduced for regions with additive-by-dominance/dominance-by-additive effects. This latter observation is partially due to the confounding between the direct dominance effect and the additive-by-dominance/dominance-by-additive epistatic effects as observed in different estimates among different chains in some cases. This suggests that several combinations of TRD estimates maximize the likelihood of the data, as mentioned before, and therefore, it is more difficulty to estimate their effects. Moreover, regions with additive-by-additive effects showed the clearest patterns of epistatic effects. One of the regions with an estimated αα_e_ = 0.99 (standard deviation = 0.00015, log_10_(BF_αα_) = 4,880.73) displayed an over-representation of 8,054 offspring (40.04%; 20,117 observed vs. 12,063 expected) for AABB and 14,800 (30.29%; 48,867 observed vs. 34,067 expected) for aaBB, whereas an under-representation of 5,567 (49.80%; 5,633 observed vs. 11,179 expected) for aaBB and 4,227 (66.85%; 2,100 observed vs. 6,323 expected) for AAbb (Figure 1A, Supplementary Material S3). On the other hand, regarding the dominance-by-dominance effect, a pair of regions with δδ_e_ = −0.99 (standard deviation = 0.00004, log_10_(BF_δδ_) = 16,219.5) showed an under-representation of 14,338, 14,079, 25,459 and 24,814 for AaBB, AABb, aaBb and Aabb, respectively, and an over-representation of 17,864, 1, 37,346, 9 and 29,853 for AABB, aaBB, AaBb, AAbb and aabb, respectively, following the parameterization for dominance-by-dominance effects (Figure 1B, Supplementary Material S3). Those examples are the simplest cases with clear epistatic TRD patterns, but it must be taken into consideration that most regions have several direct and epistatic effects with different statistical significance as evidence of the complexity of the epistatic phenomenon in the genome of cattle.

Furthermore, identification of SNPs interacting with several physically linked SNPs supports the relevance of the epistasis phenomenon. This observation is potentially explained by the LD, where one individual or different physically linked SNPs (i.e., a genomic region/locus) interact with one or different physically linked SNPs, supporting the importance of the epistatic interaction found. For this reason, only the pair of SNPs with the highest BF were maintained as the best candidate regions when one single locus interacted with several physically linked SNPs. On the other hand, an individual SNP interacting with several not linked SNP were also found. This latter case might potentially suggest a multiple interaction involving different unlinked loci (of order 3 or more).

### 4.2 Allelic epistatic transmission ratio distortion

The preliminary scan with the allelic model was implemented only in pairs of loci with already identified SNP with direct TRD effect in order to target possible epistatic interaction explaining the incomplete penetrance (deviation) of single-locus TRD. The criteria implemented in the simplified method were used to ensure more TRD was explained with epistatic interaction and that the observed TRD of the individual SNP was captured by an artificial haplotype. Explicitly, the restriction of an equal or greater number of under-/over-represented offspring was used to guarantee that the under-/over-represented offspring generated from a specific parent with a particular SNP allele corresponds to a single artificial haplotype (gamete) on those parents. Note that the linkage of specific alleles in a single gamete could be a result of selection or random effects, as it is known by theory that selection or genetic drift could favor combinations of alleles and consequently induce LD at physically unlinked loci (Bulmer, 1971; Ohta, 1982). However, by using trios of parent-offspring genotypes with offspring genotyped at early age, the issue of selection potentially was minimized, and the detected regions are expected to be associated with the reproduction cycle. In this case, under the hypothesis that the partial lethality of one allele at the first locus is linked to a specific allele on the second paired locus, this methodology could allow more insights to be gained in understating the penetrance of lethal alleles when knowing the genotype of the implicated loci. For instance, the variation of penetrance explained by the interaction could be due to some functional interaction of some unfavorable allelic combinations when inherited together in the genome of the progeny and probably affecting the fertility of parents and/or viability of embryos/offspring.

On the other hand, it is important to mention that, as the analyses are restricted to SNPs with single-locus TRD in which the majority had low frequencies (Id-Lahoucine, 2020), the multiple test combination with all other SNPs could easily generate allelic epistasis in some of them by chance. Indeed, SNPs interacting with several SNPs were observed. Specifically, only 11 pairs of SNPs had one single interaction whereas the maximum number of SNPs observed interacting with one individual SNP was 866. Even though it is plausible to assume that there are multiple interactions between several loci, these multiple interactions must be taken with caution given the low frequencies of the analyzed SNPs (from the 2,962 used SNPs, 1,424 and 950 had frequency <0.05 and 0.01, respectively). However, given the analysis were restricted to candidate regions (with low frequencies in most cases), the pair of loci with the highest BF was considered as the best candidate region with epistatic TRD, when the same SNP was found interacting with several SNPs.

The results of the analysis using the full model including simultaneously both direct and epistatic effects supported the previous findings. First, this emphasizes the effectiveness of the simplified approach to largely reduce the computational time. Second, in comparison to the simplified method, the epistatic effects observed in one artificial haplotype were also observed in all (3) or some of the heterozygous pairwise combinations including the artificial haplotype identified. An example of this was an artificial haplotype (aB) with a TRD magnitude of 0.37 (log_10_(BF) = 769.12) that, using biallelic-haplotype method, showed a TRD of −0.78 (log_10_(BF) = 351.96), −0.67 (log_10_(BF) = 216.05) and 0.74 (log_10_(BF) = 192.72) for β_AB/aB_, β_Ab/aB_ and β_aB/ab_, respectively, whereas keeping β_A_ = −0.31 (log_10_(BF) = 471.30) and β_B_ = 0.29 (log_10_(BF) = 328.74) for direct effect. The number of regions which showed clear epistatic effects in all heterozygous pairwise combinations including the artificial haplotype, was 29. Nevertheless, in some cases, epistatic TRD was also observed in heterozygous pairwise combinations with other artificial haplotype alleles. This result adds difficulties in understanding epistatic TRD, but could be explained by the different linkage with the causal mutations among families, higher order of epistasis interactions, and does not rule out the possibility of involvement of specific interactions between the artificial haplotypes. A total of 17 regions were identified when only pairs of regions displaying epistatic TRD for a single artificial haplotype were considered (Table 3).

Moreover, we observed that the magnitude obtained from single-locus analyses were similar (mostly identical) to those obtained from the epistatic allelic model (direct effects) when both direct and epistatic TRD effect were simultaneously estimated. This interesting behavior support the robustness and the accuracy of allelic model, where basically the model it tried to track the inheritance of alleles from parents to offspring independently of its source. It is also important to mention that a similar behavior was already reported in single-locus TRD analyses, thus highlighting the robustness of allelic model when comparing both allelic and genotypic model (Id-Lahoucine, 2020; Id-Lahoucine et al., 2022). Thus, this latter suggests that epistatic TRD could generate signals of single-locus TRD (as expected) and consequently could make difficult to differentiate between the origins of single-locus TRD in some cases. However, it is important to emphasize that the likelihood ratio between the full and null TRD models exhibited values up to 10^1448.11^ (minimal observed was 10^12.71^). More specifically, the epistatic parameters seems to be very importance as the likelihood ratio between the full model and an alternative model without epistatic parameters exhibited values up to 10^1,261.89^ (minimal 2,630.27), supporting the importance of the epistatic TRD phenomenon in the data.

Finally, it is important to highlight that not always a disadvantage was observed when a specific artificial haplotype was transmitted, but also artificial haplotype alleles with positive effects were found. In this sense, one single artificial haplotype with a negative TRD was observed, associated with an under-represented offspring and potentially lethality of the offspring of carrier parent, whereas the alternative artificial haplotypes had positive effects. In contrast, when one single artificial haplotype had a positive effect (whereas negative or null for the remaining artificial haplotypes), it suggests the advantage of one haplotype to be transmitted over other haplotypes. This could be due to an increase in fitness when an allele is paired with a specific allele at another locus. In addition, it could be hypothesized that these regions with positive selection are more related to biological processes acting before fertilization, where gametes with specific allele combinations have a preferential transmission to the next-generation.

### 4.3 Recessive epistatic transmission ratio distortion

Absence (or depletion) of individuals with two-locus genotypes in the homozygous statue were found across the Holstein genome, suggesting multiple variants with recessive epistatic TRD pattern. The number of candidate pairs of loci were 56, with 23 of them having the maximum number of expected homozygous offspring, but none of them being observed. It is important to mention that Holstein haplotype 3 was identified initially by VanRaden et al. (2011) with only 7 non-observed homozygous offspring from heterozygous sires in combination with heterozygous maternal grandsires. These findings provide evidence of regions carrying deleterious mutations that are expressed when presented together in a homozygous state, potentially producing lethality.

### 4.4 Quantitative trait loci and epistatic transmission ratio distortion

To support TRD findings, several quantitative trait loci (QTL) from the Cattle QTL database (CattleQTLdb, www.animalgenome.org/cgi-bin/QTLdb/BT/index) were found to overlap with epistatic TRD regions. Among them, the pair of SNPs BTA1:36209316 and BTA21:26405185 were reported with QTL for reproduction traits such as gestation length (Maltecca et al., 2008) and first service to conception traits (Kiser et al., 2019). Cole et al. (2011) reported QTL for both Calving ease and Stillbirth for BTA23:1674622 and BTA28:2233574. Other pairs of SNPs (BTA 2:27724930 and BTA 7:10835967) were also found with QTL for calving ease and stillbirth by Cole et al. (2011); Müller et al. (2017). In addition, QTL for the interval from first to last insemination and non-return rate (Müller et al., 2017), interval to first estrus after calving (Liu et al., 2017) and conception rate at first service (Galliou et al., 2020) were also found for BAT4:85759993 and BAT21:37978844. For the particular pair “BAT7:10835967 and BAT8:101251865” we find the existence of QTL for the interval from first to last insemination (Zhang et al., 2019) and daughter pregnancy rate (Cole et al., 2011). The SNP BAT7:10835967 also interacted with BAT10:29761646 and where QTL were reported for calving ease, daughter pregnancy rate and stillbirth by Cole et al. (2011), calving ease by Müller et al. (2017), interval to first estrus after calving by Liu et al. (2017) and conception rate at first service by Galliou et al. (2020). Moreover, the same SNP BAT7:10835967 interacted with BAT24:33863680 and both presented a QTL for stillbirth by Thomasen et al. (2008) and Müller et al. (2017). All the results supported the reliability of epistatic TRD findings, however, further research investigating more in-depth the genomic regions with epistatic TRD concerning their biological and functional implications are needed.

## 5 Conclusion

Our results aimed to elucidate the prevalence and patterns of epistatic transmission ratio distortion across the Holstein genome. Different epistatic TRD patterns were observed. Using genotypic models, 7, 19 and 6 pairs of SNPs with additive-by-additive, additive-by-dominance/dominance-by-additive and dominance-by-dominance effects were identified with decisive evidence, respectively. The allelic TRD model revealed 17 pairs of SNPs that offered more insights into understating the penetrance of single-locus lethal alleles, providing a more exact probability of lethality when the genotype of both implicated loci is taken into consideration. Scanning for the depletion of individuals carrying double homozygous genotypes for unlinked loci compared to expected, revealed 56 pairs of SNPs with a recessive epistatic TRD pattern. Additionally, the detection of epistatic TRD on pairs of SNPs with QTL for reproductive traits supported the reliability of epistatic TRD findings. Finally, in this study, we demonstrated candidate genomic regions harboring epistatic interactions with potential biological implications in economically important traits, such as reproduction.

## Data Availability

The data analyzed in this study is subject to the following licenses/restrictions: Data that support the findings of this study are available from the Canadian Dairy Network (a member of Lactanet, Guelph, Ontario, Canada) upon reasonable request to the corresponding author, but restrictions apply to the availability of these data, which were used under a license of a material transfer agreement for the current study, and thus are not publicly available. Requests to access these datasets should be directed to Angela Cánovas, acanovas@uoguelph.ca.
