# Supplementary material for: Parent-offspring genotyped trios unravelling genomic regions with gametic and genotypic epistatic transmission bias on the cattle genome
Source: Front Genet. 2023 Apr 6;14:1132796. doi: 10.3389/fgene.2023.1132796 (PMC10117652; doi:10.3389/fgene.2023.1132796)
Supplement: Supplementary file 1 [file DataSheet1.zip › Supplementary Material/Supplementary Material 1.pdf]

Supplementary Material

Parent-offspring genotyped trios unravelling genomic regions with gametic and genotypic epistatic transmission bias on the cattle genome

Samir Id-Lahoucine, Joaquim Casellas, Filippo Miglior, Flavio S. Schenkel, Angela Cánovas*

*** Correspondence:** Angela Cánovas: acanovas@uoguelph.ca

Supplementary Material 1. Number of animals genotyped for each density SNP array and common SNPs used for imputation.

SNP array	Number of SNPs	Number of individuals	Number of common SNPs for imputation
BovineSNP50 BeadChip[1]	55,647	43,710	47,910
Bio-Gensys BGBoviSN[2]	57,513	28	45,399
BovineHD BeadChip[1]	777,962	19	43,708
Medium Density V.2[4]	60,914	2,111	42,735
Medium Density[4]	56,955	560	42,160
GGP Bovine 150K Array[3]	139,914	8,513	39,780
Low Density[4]	11,404	19,711	26,963
GGP Bovine 50K[3]	47,850	4,328	14,546
Low Density V.4[4]	18,815	52,003	11,190
Low Density V.5[4]	27,780	6,589	10,886
Genomic Profiler LD[3]	26,151	62,091	10,737
Genomic Profiler-HD[3]	77,068	19,920	9,994
Genomic Profiler LD V.4[3]	30,112	29,665	9,807
Low Density V.2[4]	17,619	61,572	8,152
Genomic Profiler-Super LD[3]	19,809	41,469	7,956
Genomic Profiler[3]	8,762	23,454	7,682
BovineLD BeadChip V.1.1[1]	6,912	4,529	6,607
BovineLD BeadChip[1]	6,909	36,907	6,605
EuroG10K[1]	9,072	106	6,605
GGP Bovine 7K[3]	7,083	9,249	6,032
GGP Bovine 9K[3]	8,984	1,846	6,004

[1]Illumina, Inc., San Diego, CA.

[2]Affymetrix, Santa Clara, CA.

[3]Gene Seek, Lincoln, NE.

[4]Zoetis, Florham Park, NJ.
