# Supplementary material for: Parent-offspring genotyped trios unravelling genomic regions with gametic and genotypic epistatic transmission bias on the cattle genome
Source: Front Genet. 2023 Apr 6;14:1132796. doi: 10.3389/fgene.2023.1132796 (PMC10117652; doi:10.3389/fgene.2023.1132796)
Supplement: Supplementary file 1 [file DataSheet1.zip › Supplementary Material/Supplementary Material 2.pdf]

Supplementary Material

Parent-offspring genotyped trios unravelling genomic regions with gametic and genotypic epistatic transmission bias on the cattle genome

Samir Id-Lahoucine, Joaquim Casellas, Filippo Miglior, Flavio S. Schenkel, Angela Cánovas*

*** Correspondence:** Angela Cánovas: acanovas@uoguelph.ca

Supplementary Material 2. Informative matings grouped with the expected proportions in the offspring generation.

Matings (parent x parent genotypes)	AABB	AaBB	aaBB	AABb	AaBb	aaBb	AAbb	Aabb	aabb
AaBb × AaBb	1	2	1	2	4	2	1	2	1
AaBB × AaBB	4	8	4	0	0	0	0	0	0
Aabb × Aabb	0	0	0	0	0	0	4	8	4
AABb × AABb	4	0	0	8	0	0	4	0	0
aaBb × aaBb	0	0	4	0	0	8	0	0	4
AaBB × AaBb; AaBb × AaBB	2	4	2	2	4	2	0	0	0
Aabb × AaBb; AaBb × Aabb	0	0	0	2	4	2	2	4	2
AaBB × Aabb; Aabb × AaBB	0	0	0	4	8	4	0	0	0
AABb × AaBb; AaBb × AABb	2	2	0	4	4	0	2	2	0
aaBb × AaBb; AaBb × aaBb	0	2	2	0	4	4	0	2	2
AaBB × AABB; AABB × AaBB	8	8	0	0	0	0	0	0	0
AaBB × aaBB; aaBB × AaBB	0	8	8	0	0	0	0	0	0
Aabb × Aabb; AAbb × Aabb	0	0	0	0	0	0	8	8	0
Aabb × aabb; aabb × Aabb	0	0	0	0	0	0	0	8	8
AABb × aaBb; aaBb × AABb	0	4	0	0	8	0	0	4	0
AABb × AABB; AABB × AABb	8	0	0	8	0	0	0	0	0
aaBb × aaBB; aaBB × aaBb	0	0	8	0	0	8	0	0	0
AABb × Aabb; AAbb × AABb	0	0	0	8	0	0	8	0	0
aaBb × aabb; aabb × aaBb	0	0	0	0	0	8	0	0	8
AABb × aaBB; aaBb × AABB; aaBB × AABb; AABB × aaBb	0	8	0	0	8	0	0	0	0
AABb × aabb; aaBb × Aabb; AAbb × aaBb; aabb × AABb	0	0	0	0	8	0	0	8	0
AaBB × AABb; AABb × AaBB; AaBb × AABB; AABB × AaBb	4	4	0	4	4	0	0	0	0
AaBB × aaBb; aaBb × AaBB; AaBb × aaBB; aaBB × AaBb	0	4	4	0	4	4	0	0	0
Aabb × AABb; AABb × Aabb; AaBb × Aabb; AAbb × AaBb	0	0	0	4	4	0	4	4	0
Aabb × aaBb; aaBb × Aabb; AaBb × aabb; aabb × AaBb	0	0	0	0	4	4	0	4	4
AaBB × Aabb; Aabb × AABB; AABB × Aabb; AAbb × AaBB	0	0	0	8	8	0	0	0	0
AaBB × aabb; Aabb × aaBB; aaBB × Aabb; aabb × AaBB	0	0	0	0	8	8	0	0	0
